# Limited impact of cancer-derived gangliosides on anti-tumor immunity in colorectal cancer

**DOI:** 10.1093/glycob/cwae036

**Published:** 2024-05-24

**Authors:** Irene van der Haar Àvila, Tao Zhang, Victor Lorrain, Florance de Bruin, Tianne Spreij, Hitoshi Nakayama, Kazuhisa Iwabuchi, Juan J García-Vallejo, Manfred Wuhrer, Yvette van Kooyk, Sandra J van Vliet

**Affiliations:** Department of Molecular Cell Biology and Immunology, Amsterdam UMC location Vrije Universiteit Amsterdam, de Boelelaan 1117, 1081 HZ Amsterdam, the Netherlands; Cancer Center Amsterdam, Cancer Biology and Immunology, Amsterdam, the Netherlands; Cancer Immunology, Amterdam institute for Immunology and Infectious Diseases, Amsterdam, the Netherlands; Center for Proteomics and Metabolomics, Leiden University Medical Center, Albinusdreef 2, 2333 ZA Leiden, the Netherlands; Department of Molecular Cell Biology and Immunology, Amsterdam UMC location Vrije Universiteit Amsterdam, de Boelelaan 1117, 1081 HZ Amsterdam, the Netherlands; Department of Molecular Cell Biology and Immunology, Amsterdam UMC location Vrije Universiteit Amsterdam, de Boelelaan 1117, 1081 HZ Amsterdam, the Netherlands; Department of Molecular Cell Biology and Immunology, Amsterdam UMC location Vrije Universiteit Amsterdam, de Boelelaan 1117, 1081 HZ Amsterdam, the Netherlands; Graduate School of Health Care and Nursing, Laboratory of Biochemistry, Juntendo University, 2-5-1 Takasu Urayasu-shi, Chiba, 279-0023, Japan; Graduate School of Health Care and Nursing, Laboratory of Biochemistry, Juntendo University, 2-5-1 Takasu Urayasu-shi, Chiba, 279-0023, Japan; Department of Molecular Cell Biology and Immunology, Amsterdam UMC location Vrije Universiteit Amsterdam, de Boelelaan 1117, 1081 HZ Amsterdam, the Netherlands; Cancer Center Amsterdam, Cancer Biology and Immunology, Amsterdam, the Netherlands; Cancer Immunology, Amterdam institute for Immunology and Infectious Diseases, Amsterdam, the Netherlands; Center for Proteomics and Metabolomics, Leiden University Medical Center, Albinusdreef 2, 2333 ZA Leiden, the Netherlands; Department of Molecular Cell Biology and Immunology, Amsterdam UMC location Vrije Universiteit Amsterdam, de Boelelaan 1117, 1081 HZ Amsterdam, the Netherlands; Cancer Center Amsterdam, Cancer Biology and Immunology, Amsterdam, the Netherlands; Cancer Immunology, Amterdam institute for Immunology and Infectious Diseases, Amsterdam, the Netherlands; Department of Molecular Cell Biology and Immunology, Amsterdam UMC location Vrije Universiteit Amsterdam, de Boelelaan 1117, 1081 HZ Amsterdam, the Netherlands; Cancer Center Amsterdam, Cancer Biology and Immunology, Amsterdam, the Netherlands; Cancer Immunology, Amterdam institute for Immunology and Infectious Diseases, Amsterdam, the Netherlands

**Keywords:** colorectal cancer, glycosphingolipids, sialylation, ST3Gal5

## Abstract

Aberrant glycosylation is a key mechanism employed by cancer cells to evade immune surveillance, induce angiogenesis and metastasis, among other hallmarks of cancer. Sialic acids, distinctive terminal glycan structures located on glycoproteins or glycolipids, are prominently upregulated across various tumor types, including colorectal cancer (CRC). Sialylated glycans modulate anti-tumor immune responses through their interactions with Siglecs, a family of glycan-binding receptors with specificity for sialic acid-containing glycoconjugates, often resulting in immunosuppression. In this paper, we investigated the immunomodulatory function of ST3Gal5, a sialyltransferase that catalyzes the addition of α2-3 sialic acids to glycosphingolipids, since lower expression of ST3Gal5 is associated with better survival of CRC patients. We employed CRISPR/Cas9 to knock out the *ST3Gal5* gene in two murine CRC cell lines MC38 and CT26. Glycomics analysis confirmed the removal of sialic acids on glycolipids, with no discernible impact on glycoprotein sialylation. Although knocking out ST3Gal5 in both cell lines did not affect in vivo tumor growth, we observed enhanced levels of regulatory T cells in CT26 tumors lacking ST3Gal5. Moreover, we demonstrate that the absence of ST3Gal5 affected size and blood vessel density only in MC38 tumors. In summary, we ascertain that sialylation of glycosphingolipids has a limited influence on the anti-tumor immune response in CRC, despite detecting alterations in the tumor microenvironment, possibly due to a shift in ganglioside abundance.

## Introduction

Sialic acids (Sias) are a family of negatively charged, nine-carbon monosaccharides present at the outermost end of mammalian *N*-linked and *O*-linked glycoproteins or bound to lipid-based glycoconjugates. They decorate the surface of multiple cell types and are involved in many cellular processes, including cell–cell interactions, communication, and cell signaling. Sias can be found in different linkages, namely α2-3, α2-6 and α2-8 linked Sias, depending on their attachment to underlying residues (Lübbers et al. 2018). They are added to glycoproteins or glycolipids by different enzymes termed amongst others α2-3 sialyltransferases (ST3Gals) and α2-6 sialyltransferases (ST6Gals) (Varki et al. 2022). Besides, Sias can serve as ligands for Sialic acid-binding immunoglobulin-type lectin (Siglec) receptors or as binding sites for various pathogens and toxins (Varki 2008; Varki et al. 2022).

In numerous tumors, including colorectal cancer (CRC), the amount of sialoglycans covering the cell surface is enhanced, this being considered a malignant feature of cancer cells (Fuster and Esko 2005; Büll et al. 2014; Pinho and Reis 2015; Büll et al. 2018; Rodríguez et al. 2018). Hypersialylation of tumors is associated with poor clinical outcome of patients, suggesting that specific sialyltransferases or the receptors for sialylated glycans, might be potential therapeutic targets (Büll et al. 2014; Zhou et al. 2020). The upregulation of specific sialyltransferases or the downregulation of sialidases, and thus the accumulation of Sias, is associated with invasion, migration, epithelial-mesenchymal transition (EMT) and metastasis. Moreover, the coating of Sias on tumor cells can act as “self-associated molecular patterns” (SAMPs), helping tumors to escape immune surveillance through the interaction with Siglec receptors (Varki 2011; Bartish et al. 2020; Varki et al. 2022). Most Siglec receptors possess an immunoreceptor tyrosine-based inhibitory motif (ITIM) and act in a similar manner as other immune checkpoint molecules, such as PD-1 (Lübbers et al. 2018; Stanczak and Läubli 2023). So, upon engagement with sialylated glycans, they inhibit the function of a variety of innate immune cells that express the Siglec receptors. Previous studies have shown that tumor-associated Sias inhibited NK cell activation by binding to Siglec-7 and Siglec-9 (Hudak et al. 2014; Jandus et al. 2014). Furthermore, increased sialylation on pancreatic tumor cells drives tumor-associated macrophage (TAM) differentiation also via signaling of Siglec-7 and Siglec-9 receptors (Rodriguez et al. 2021). TAMs additionally express Siglec-15, and the interaction of Siglec-15 with the Sialyl-Tn antigen on tumor cells can enhance the production of the anti-inflammatory cytokine TGF-β by macrophages (Takamiya et al. 2013).

The Sia-Siglec interaction not only impacts innate immunity, but also affects the functionality and activation of adaptive immune cells. For example, the binding of sialylated antigens to Siglec receptors on dendritic cells (DCs) can decrease effector T cell differentiation favoring the development of regulatory T cells (Tregs) in vivo (Perdicchio et al. 2016b). Also, hypersialylation on tumor cells promoted tumor growth and was likewise associated with the induction of Tregs and a reduction of cytotoxic T cells (Perdicchio et al. 2016a). While T cells generally have low expression of Siglecs, recent studies indicate that expression of Siglecs is elevated in tumor-infiltrating lymphocytes (TILs) (Stanczak et al. 2018; Haas et al. 2019).

Given the role of tumor sialylation in the establishment of an immunosuppressive tumor microenvironment (TME), the sialic acid-Siglec axis is now regarded as a “glyco”- immune checkpoint that can be targeted therapeutically to improve anti-tumor immunity (Bärenwaldt and Läubli 2019; Gray et al. 2020). Indeed, disrupting the Sia-Siglec pathway improves tumor immunesurveillance through enhanced infiltration of CD8^+^ T cells and a delay in tumor growth (Perdicchio et al. 2016a; Stanczak et al. 2018; Gray et al. 2020; Boelaars et al. 2023). In a breast cancer model, enhanced anti-tumor immunity after desialylation was dependent on expression of the Siglec-E (murine orthologue of Siglec-9) receptor found on tumor-infiltrating myeloid cells (Gray et al. 2020).

ST3Gal5, also known as GM3 synthase, is the sialyltransferase responsible for adding α2-3-linked Sias to lactosylceramide (LacCer) to initiate the biosynthesis of several ganglioside species and forms the precursor for complex glycosphingolipids (GSLs), such as *a*-series and *b*-series gangliosides and for GM4, by using LacCer and galactosylceramide (GalCer) as substrates, respectively (Inokuchi and Uemura 2014; Uemura et al. 2014). Gangliosides, together with cholesterol and other transmembrane signaling proteins, can form membrane microdomains, also known as lipid rafts (Regina Todeschini and Hakomori 2008). These ganglioside-based lipid rafts are important for modulating cell signaling and are thereby also involved in malignancy (Simons and Gerl 2010; Inokuchi and Uemura 2014). Gangliosides also increase the cellular responsiveness of endothelial cells to vascular endothelial growth factor (VEGF), a key factor for promoting the formation of new blood vessels (Zhou et al. 2020). In addition, gangliosides are also shed by tumor cells or are released by extracellular vesicles into circulation, where they remodel the TME and facilitate tumor cell metastasis (Nejatie et al. 2023; van der Haar Àvila et al. 2023).

Although the exact function of ST3Gal5 in disease is not fully understood yet, some findings indicate the importance of GM3 synthase in neurological and mitochondrial disorders (Simpson et al. 2004; Fragaki et al. 2013; Lee et al. 2016; Indellicato et al. 2019). In addition, abnormal expression of ST3Gal5 is found in multiple types of cancers. However, its function in cancer is still unclear as multiple studies show opposite roles for this enzyme during tumor development and progression. For instance, in bladder and lung cancer, expression of ST3Gal5 was lower in the tumor compared to adjacent normal tissues, which correlated with poor prognosis and reduced patient survival (Ouyang et al. 2020; Zhang et al. 2023). Contrary, ST3Gal5 was significantly overexpressed in renal cell carcinoma tumors and it was associated with poor clinical outcome (Liu et al. 2022), reflecting the different roles of ST3Gal5 depending on the tumor type. In other studies, higher levels of ST3Gal5 were observed in vascular endothelial cells, immune cells as well as cancer stem cells, marking the controversial and perhaps multi-level role of this enzyme in cancer (Liang et al. 2013; Dewald et al. 2018; Suzuki et al. 2021).

Aberrant expression of ST3Gal5 in the TME consequently leads to alterations in the production and presence of the gangliosides repertoire. Gangliosides, like sialylated glycoproteins, serve as ligands for Siglec receptors. For instance, GM3 has been shown to bind to Siglec-9, expressed on myeloid cells, NK cells and a subset of T cells, thereby exerting an inhibitory influence on those immune cells. Hence, the interaction between gangliosides and Siglecs might play a crucial role in modulating anti-tumor immunity. Moreover, gangliosides can also modulate immune responses by inducing T cell apoptosis and/or interfering with cytokine production (van der Haar Àvila et al. 2023).

In renal cell carcinoma, high expression of ST3Gal5 was correlated to infiltration of exhausted CD8^+^ T cells (Liu et al. 2022). However, to what extent sialylated GSLs mechanistically modulate immune cells in the TME and contribute to immune evasion, still remains unexplored. Prior research clearly demonstrated the involvement of Sias on anti-tumor immunity, yet none of these studies made a distinction between sialylated glycoproteins or the influence of sialylated lipid species. It is well established that CRC tumor cells are heavily glycosylated, carrying high levels of Sias, sustaining an immunosuppressive microenvironment (Holst et al. 2015; Venkitachalam et al. 2016). Also, recurrence of CRC is correlated with a distinct glycosylation profile, illustrating the relevance of tumor sialylation in CRC (Lenos et al. 2015). Therefore, in this paper we evaluated the role of ST3Gal5 in regulating the anti-tumor immune response in CRC. To our knowledge, this is the first study exploring the role of ST3Gal5 in this tumor type. To investigate this, we modified the sialylation pathway of GSLs in two murine CRC models (MC38 and CT26 cells) by knocking out the *ST3Gal5* gene using CRISPR/Cas9 genome engineering. We successfully reduced the levels of sialylated GSLs in the MC38- and CT26-ST3Gal5 KO cells. This effect was paired with an increase of non-sialylated *o*-series gangliosides and other neutral GSLs. We further demonstrate that sialylated GSLs have little impact on tumor growth and immune surveillance in CRC, in contrast to previous studies where reduced overall sialylation improved anti-tumor immunity.

## Results

### High *ST3Gal5* gene expression is associated with poor prognosis in CRC

**Fig. 1:**
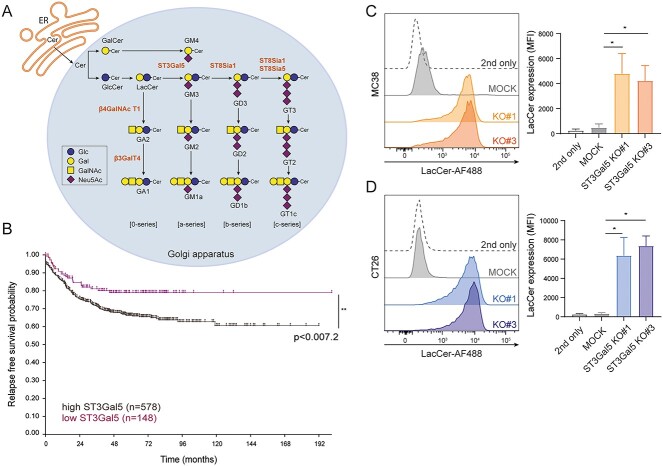
Characterization of *ST3Gal5* in CRC and generation of ST3Gal5 KO cell lines. A) Schematic representation of the ganglioside biosynthesis pathway. B) *ST3Gal5* gene expression data from a CRC cohort (*n* = 726) was analyzed by stratifying the patients in two groups with either low *ST3Gal5* gene expression (*n* = 148) or high *ST3Gal5* gene expression (*n* = 578). The relapse free-survival was evaluated (^*^^*^P ≤ 0.01). C–D) Removal of sialylated GSLs through CRISPR/Cas9 KO using two guideRNAs targeting distinct regions in the *ST3Gal5* gene. Both guideRNAs result in increased LacCer levels in MC38 (C) and CT26 cells (D) as measured by flow cytometry. MOCK-transfected cells were used as a negative control. Data are representative of four independent experiments showing the mean fluorescence intensities (MFI). Mean ± SD; unpaired non-parametric *t-*test (^*^P ≤ 0.05).

In the Golgi apparatus the ST3Gal5 enzyme catalyzes the conversion of LacCer into GM3, which is the precursor for the formation of *a*-series, *b-*series and *c-*series gangliosides. In addition, ST3Gal5 can also sialylate GalCer to produce the ganglioside GM4 (Fig. 1A). ST3Gal5 and its ganglioside products are known to be involved in many cellular signaling pathways in health and in disease; however, their immunomodulatory role in CRC has not been thoroughly investigated. Therefore, we first assessed whether expression of *ST3Gal5* is associated clinical outcome in CRC patients. We analyzed the combined cohorts of CRC patients described by Guinney *et al.* (*n* = 726) using the R2 Genomics Analysis and Visualization platform (Guinney et al. 2015). We stratified the CRC patients according to their *ST3Gal5* gene expression into two groups, *ST3Gal5* low (*n* = 148) or *ST3Gal5* high (*n* = 578) patients, and evaluated their relapse-free survival. In line with other studies, in hepatocellular and renal cell carcinoma (Cai et al. 2017; Liu et al. 2022), low *ST3Gal5* expression correlated with a higher relapse-free survival in CRC patients, suggesting that a lack of gangliosides is more beneficial in these types of cancer (Fig. 1B).

### *ST3Gal5* KO results in complete reduction of sialylated gangliosides

To investigate the role of ST3Gal5 in tumor immunogenicity, we knocked out the *ST3Gal5* gene in two different murine CRC cell lines: MC38 and CT26; both of them having comparable expression of *ST3Gal5* (Fig. S1A) We designed two different CRISPR/Cas9 gRNAs, targeting distinct regions in the *ST3Gal5* gene. As ST3Gal5 catalyzes the first step in ganglioside synthesis (Fig. 1A), abrogating its expression should abolish the addition of Sias and result in increased LacCer and GalCer levels at the cell membrane. Indeed, independent of the gRNA used, both *ST3Gal5* knockouts (MC38/CT26-ST3Gal5 KO) displayed an overexpression of LacCer on the cell surface, as measured by flow cytometry (Fig. 1C and D). Additionally, we confirmed the loss of ST3Gal5 at mRNA level by qRT-PCR (Fig. S1B), using primers that specifically anneal to the gRNA-targeted sequence and thus only amplify the WT construct. As a control, we transfected the cells with an empty CRISPR/Cas9 construct (MC38/CT26-MOCK). Similar to the CT26-WT and MC38-WT cell lines (Fig. S1C and D), the MOCK cell lines did not express LacCer (Fig. 1C and D). In addition, we confirmed that overall protein sialylation was not affected, as the plant lectins *Maackia amurensis I* and *II* (MAL-I and MAA-II) still showed binding to α2-3 Sias on *N*-glycans and *O*-glycans, respectively (Fig.S1C and D). However, both KO#3 cell lines displayed a slight reduction in MAL-I reactivity, that could be attributed to binding of the lectin to gangliosides or a reduction in α2-3 sialylation of glycoproteins.

**Fig. 2:**
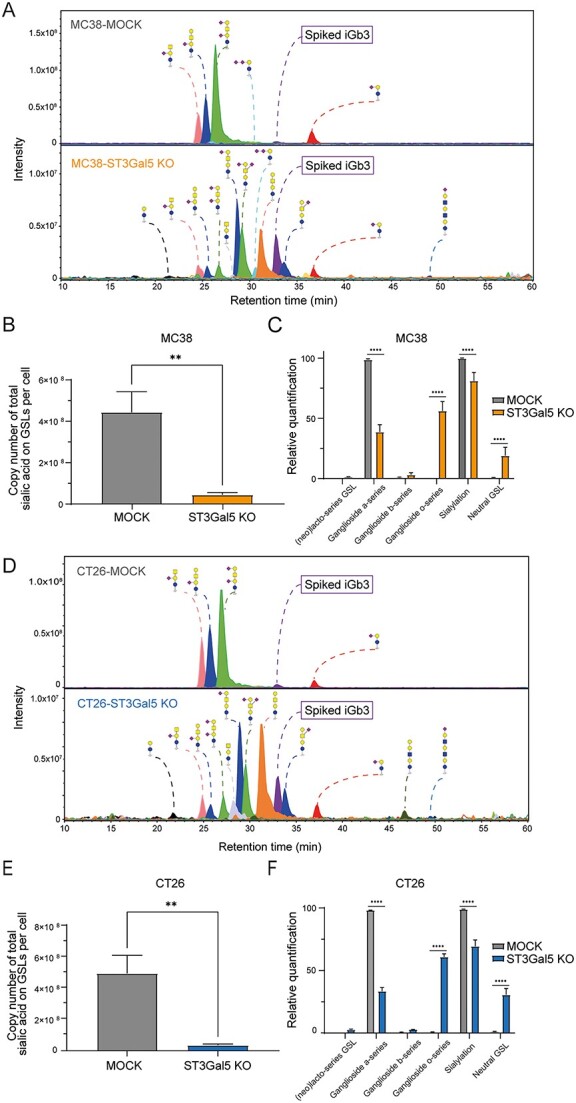
Reduced levels of sialylated gangliosides upon *ST3Gal5* gene knockout in MC38 and CT26 cells. Combined extracted ion chromatography analysis of GSL-glycans in MC38-MOCK and MC38-ST3Gal5 KO (A) or CT26-MOCK and CT26-ST3Gal5 KO (D) cells. Spiked iGb3 is used as an internal normalization control. Data are representative of three independent replicates. B and E) Total copy number of Sias carried by GSLs per cell performed in triplicates in MC38 (B) and CT26 (E) cells. Mean ± SD; unpaired non-parametric *t-*test (^*^^*^P ≤ 0.01). C and F) Relative quantification of the different types of gangliosides and total levels of sialylation in MC38-MOCK (C) and CT26-MOCK (F) compared to the respective ST3Gal5 KO cells. Mean ± SD; two-way ANOVA with multiple comparisons test (^*^^*^^*^^*^P < 0.0001).

To further confirm our flow cytometry results, we validated the glycosylation profile of our cell lines by mass spectrometry analysis (Fig. 2A and D). Both the MOCK MC38 and CT26 cell lines have a similar glycosphingolipid profile, with GD1a being the most abundant ganglioside, followed by GM1a and GM2 (Table S1). Overall, the number of total Sias in GSLs per cell is significantly reduced after knocking out *ST3Gal5* in MC38 as well as in CT26 cells (Fig. 2B and E). The glycomic profiling and quantification of the absolute abundances of GSLs indicated that *a-*series gangliosides were significantly diminished in the MC38-ST3Gal5 KO and CT26-ST3Gal5 KO cells, while the *b-*series gangliosides remained absent (Fig. 2A–D). Due to the knock-out of *ST3Gal5*, the *o*-series gangliosides and neutral non-sialylated GSLs were upregulated, which reflects alternative routes of GSL synthesis using LacCer as a substrate (Bhuiyan et al. 2016). Although the relative abundance of (neo)-lactoseries GSL was very low in both cell lines, there was a slight upregulation of those GSLs in the ST3Gal5 KO cells. More detailed information about the individual glycan structures assigned by tandem mass spectrometry (MS/MS) can be found in Supplementary Table S1. Thus, we concluded that we successfully created a complete knockout of the *ST3Gal5* gene in MC38 and CT26 cells through CRISPR/Cas9 genome engineering.

### Tumor growth in vivo is unaffected by ST3Gal5 KO

**Fig. 3:**
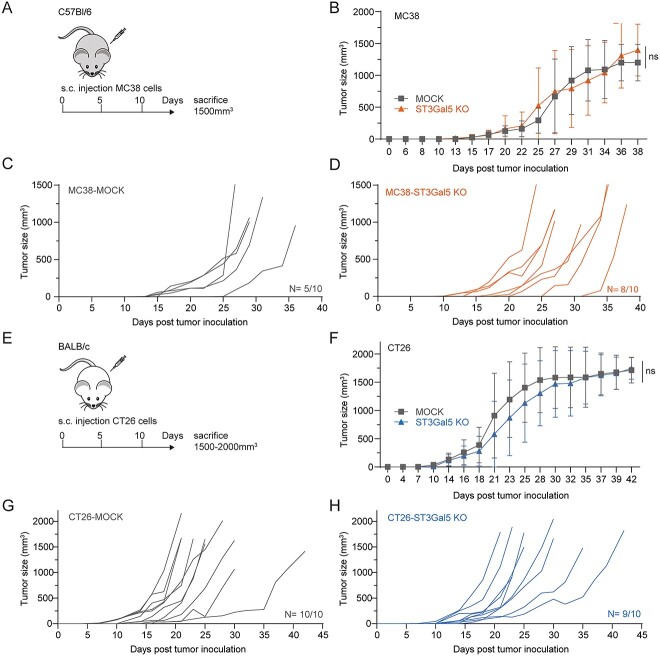
ST3Gal5 KO and MOCK tumors have similar tumor growth in vivo. A) MC38-MOCK or MC38-ST3Gal5 KO cells were injected subcutaneously into C57Bl/6 mice (*n* = 20). B–D) Tumors were measured three times per week and mice were sacrificed when the tumors reached a size around 1,500 mm^3^. Tumor growth is depicted as mean tumor size (B) or individual tumor growth curves (C and D). E) CT26-MOCK or CT26-ST3Gal5 KO cells were injected subcutaneously into BALB/c mice (*n* = 20). F–H) Mice were sacrificed when the tumors reached a size between 1,500 mm^3^ and 2,000 mm^3^. Growth of CT26 tumors was monitored three times per week and is depicted as mean tumor size (F) or individual tumor growth curves (G and H). Mean ± SD (ns, not significant).

Next, we investigated the effect of GSL desialylation on tumor progression in the two different in vivo mouse models. Manipulation of the glycosylation machinery may influence the proliferation capacity of cells. However, MC38/CT26-MOCK and MC38/CT26-ST3Gal5 KO cells exhibited identical growth rates under in vitro culture conditions (Fig. S2A and B). Subsequently, we injected MC38-MOCK and MC38-ST3Gal5 KO cells into C57Bl/6 mice and CT26-MOCK and CT26-ST3Gal5 KO cells into BALB/c mice, as these cell lines are derived from different mouse strains (Fig. 3A and E). Interestingly, MC38-MOCK and MC38-ST3Gal5 tumors grew similarly (Fig. 3B). However, only 5 of the 10 mice developed a tumor in the MC38-MOCK group suggesting a low tumor take compared to the ST3Gal5 KO group (Fig. 3C and D). Tumor growth of CT26-MOCK and CT26-ST3Gal5 KO cells was also comparable (Fig. 3F). Nevertheless, ST3Gal5 KO tumors appeared to grow slightly slower in the exponential phase, however this difference was not significant. Also, in the CT26 mouse model almost all the mice developed a tumor (Fig. 3G and H). These results indicate that the presence or absence of sialylated GSLs does not impact tumor control nor the survival of mice in both mouse tumor models (Fig. S2C and D). To evaluate overall ST3Gal5 levels in the established MC38 and CT26 tumors, we checked the gene expression of *ST3Gal5* on frozen tumor tissues with qRT-PCR. Intriguingly, we did not detect any differences between the MOCK and ST3Gal5 samples (Fig. S2E and F), indicating that other cell types, such as fibroblasts and endothelial cells within the TME still harbor significant ST3Gal5 which may explain the similarity in tumor growth in vivo.

### ST3Gal5 KO tumors harbor similar numbers of CD8 ^+^ T cells but have increased CD4^+^ Tregs compared to MOCK tumors

**Fig. 4:**
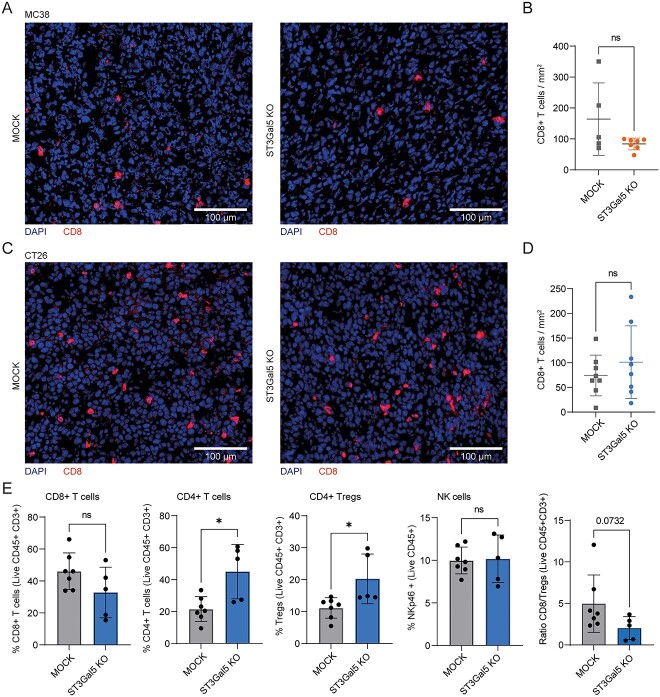
Immune profiling of MOCK and ST3Gal5 KO MC38 and CT26 tumors. A) Immunofluorescence staining of CD8 on MC38-MOCK and MC38-ST3Gal5 KO frozen tumor tissues. Representative staining of *n* = 5 and *n* = 7 samples, respectively. B) Quantification of CD8^+^ T cell counts per mm^2^ of MC38 tumor tissue analyzed. Each dot represents an individual tumor. C) Immunofluorescence staining of CD8 on CT26-MOCK and CT26-ST3Gal5 KO frozen tumor tissues. Representative staining of *n* = 8 samples. D) Quantification of CD8^+^ T cell counts per mm^2^ of CT26 tumor tissue analyzed. Each dot represents an individual tumor. E) Percentages of immune cell subsets in CT26-MOCK tumors compared to CT26-ST3Gal5 KO tumors. Samples were analyzed by flow cytometry and gated on alive, CD45^+^ immune cells (Fig. S3). Mean ± SD; unpaired non-parametric *t-*test (ns, non-significant; ^*^P ≤ 0.05).

In most solid tumors, high levels of tumor-infiltrating CD8^+^ T cells predict good clinical outcomes after treatment, while immune exclusion or an immune desert phenotype is associated with resistance to multiple therapies (Chen and Mellman 2017; Fridman et al. 2017; Mariathasan et al. 2018). In the dataset of CRC patients we analyzed, low expression of *ST3Gal5* was associated with good prognosis (Fig. 1B). Therefore, to gain more insight into the TME of MC38 and CT26 tumors and its relation with ST3Gal5 expression, we evaluated the immune cell composition in frozen tumor tissues and tumor cell suspensions. Because of the importance of CD8^+^ T cells in CRC tumor immunity (Pagès et al. 2018), we started by analyzing those cells in frozen tissues. Although two mice harboring MC38-MOCK tumors had more infiltrating CD8^+^ T cells compared to ST3Gal5 KO tumors, overall, the number of cytotoxic lymphocytes in both TMEs was comparable (Fig. 4A and B). CT26 tumors presented similar numbers of CD8^+^ T cells infiltrating the TME compared to MC38 tumors (Fig. 4C), with also no distinction between CT26-MOCK and CT26-ST3Gal5 KO tumors (Fig. 4D). Besides CD8^+^ T cells, other lymphoid populations may contribute to an altered anti-tumor immune response, such as CD4^+^ T cells, regulatory T cells (Tregs) and NK cells. We analyzed the frequencies of these immune subsets in the CD45^+^ cell compartment of CT26 tumors by flow cytometry (Fig. S3). Strikingly, CD4^+^ T cells and CD4^+^ Tregs were significantly increased in ST3Gal5 KO tumors, while percentages of CD8^+^ T cells and NK cells remained similar in both tumor types (Fig. 4E). Even though Treg levels were more elevated in ST3Gal5 KO tumors, suggestive of a more immunosuppressive TME, this was not sufficient to affect the tumor growth (Fig. 3F).

### Blood vessel density is significantly decreased in MC38-ST3Gal5 KO tumors but not in CT26 tumors

**Fig. 5:**
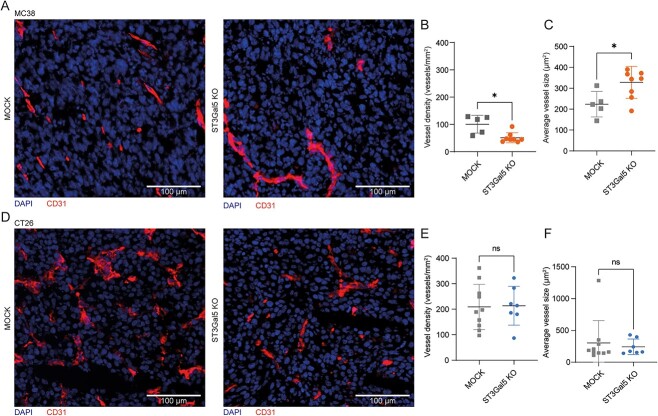
Decreased blood vessel density and increased vessels size in MC38-ST3Gal5 KO tumors. A) Immunofluorescence staining of CD31 on MC38-MOCK and MC38-ST3Gal5 KO frozen tumor tissues. Representative staining of *n* = 5 and *n* = 8 samples, respectively. B) Quantification of CD31^+^ vessels per mm^2^ of MC38 tumor tissue analyzed. Each dot represents an individual tumor. C) Average vessel size of MC38 tumors. Each dot represents an individual tumor. D) Immunofluorescence staining of CD31 on CT26-MOCK and CT26-ST3Gal5 KO frozen tumor tissues. Representative staining of *n* = 10 and *n* = 7 samples, respectively. E) Quantification of CD31^+^ vessels per mm^2^ of CT26 tumor tissue analyzed. Each dot represents an individual tumor. F) Average vessel size of CT26 tumors. Each dot represents an individual tumor. Mean ± SD; unpaired non-parametric *t-*test (ns, non-significant; ^*^P ≤ 0.05).

In a recent study, elevated levels of ST3Gal5, and thus significant amounts of gangliosides, were detected on vascular endothelial cells in human breast cancer (Suzuki et al. 2021). Tumor endothelial cells play a crucial role in blood vessel formation and immunomodulation during angiogenesis and tumor progression (Nagl et al. 2020). How ST3Gal5 can influence blood vessel formation and contribute to malignancy is not well understood yet. Therefore, we also explored the morphology (data not shown) and vasculature of MC38- and CT26-MOCK and ST3Gal5 KO tumors. Quantitative analysis of frozen tumor sections stained with CD31, a vascular endothelial marker, showed that MC38-ST3Gal5 KO tumors had reduced blood vessel formation (Fig. 5A–C). Even though the blood vessel density was clearly diminished (Fig. 5B), the average vessel size in ST3Gal5 KO tumors was significantly increased (Fig. 5C). In contrast to MC38 tumors, blood vessel size and density remained unaltered after knocking out ST3Gal5 in CT26 tumors (Fig. 5D–F). A reduction in CD31 signal in the TME of MC38-ST3Gal5 KO tumors, which correlated to blood vessel formation, could suggest a decreased angiogenesis potential in CRC tumors.

In summary, we have demonstrated that we could specifically ablate sialylated glycolipids by knocking out *ST3Gal5* in two murine cell lines, however this did not impact tumor growth in vivo. Nevertheless, ST3Gal5 KO tumors harbored higher frequencies of Tregs and had a lower blood vessel density compared to MOCK tumors, which partially correlates with our findings of the human data, showing that lower *ST3Gal5* expression is associated with a better survival in CRC patients.

## Discussion

In this paper we investigated the role of ST3Gal5 and thereby the modulation of the ganglioside biosynthesis on the anti-tumor immune response and on angiogenesis in colorectal cancer, by modifying its expression on tumor cells. In cancer, the expression levels of ST3Gal5 are altered, potentially leading to a different composition of sialylated GSLs within the TME. Tumor cells, immune cells as well as endothelial cells, all express ST3Gal5, however especially tumor cells have the ability to shed gangliosides into the tumor microenvironment (Nagafuku et al. 2012; Liu et al. 2022; van der Haar Àvila et al. 2023). In general, these sialylated gangliosides are thought to play a crucial role in modulating the immune response and may thus have a strong impact on tumor progression and metastasis (van der Haar Àvila et al. 2023). Nevertheless, how ST3Gal5, and as a consequence the generation of GSLs, affects tumor immunogenicity still remains unclear.

According to the analysis performed by Ouyang *et al.* on different CRC patient cohorts, ST3Gal5 expression is significantly lower in tumors compared to normal tissue (Ouyang et al. 2020), with lower expression also correlating with better prognosis in our CRC patient analysis (Fig. 1B). The expression levels of ST3Gal5 during malignancy, and also relative to the corresponding normal tissue, vary depending on the tumor type. For instance, in bladder cancer and lung squamous cell carcinoma, ST3Gal5 expression is lower in the tumor compared to the non-malignant adjacent tissue (Ouyang et al. 2020; Zhang et al. 2023). Contrary, ST3Gal5 is overexpressed in other tumor types, including melanoma (Ouyang et al. 2020) and breast cancer, which in the latter case is associated with a decreased overall survival of patients (Dewald et al. 2018).

In our study, we successfully knocked out ST3Gal5 by CRISPR/Cas9 technology in two different murine CRC cell lines. Both MC38 and CT26 cell lines mainly have *a*-series gangliosides and do not accumulate other type of gangliosides. So, after deletion of ST3Gal5, we observed a significant reduction of *a-*series gangliosides, resulting in LacCer accumulation and a compensatory increase of *o*-series GSLs. Thus, our results fully align with the effect on ganglioside expression observed in the ST3Gal5 KO mice from Yoshikawa *et al.* (Yoshikawa et al. 2015), whereas in fibroblasts of patients with a mutated ST3Gal5 (“Salt & Pepper” syndrome) a compensatory shift in glycoprotein sialylation could be detected when glycolipid sialylation decreases (Boccuto et al. 2014). Even though our cell lines had strongly reduced levels of sialylation only on GSLs, this did not affect tumor growth nor survival in vivo in immunocompetent mice. Opposite to our findings, overexpression of ST3Gal5 in breast cancer cells, resulted in the production of mainly *b-* and *c*-series gangliosides at the cell surface, and led to an enhanced tumor growth in immunodeficient mice (Cazet et al. 2010; Dewald et al. 2018). Likewise, ST3Gal5 in lung cancer produced *a*-series gangliosides resulting in inhibition of cell migration, invasion, and metastasis (Zhang et al. 2023). Therefore, we postulate that the effect of ST3Gal5 on anti-tumor immunity could be tumor type-dependent, since the composition of gangliosides as well as how they modulate the immune response could be different among cancer types. Moreover, in CRC cells themselves, the amount of gangliosides may be limited and therefore not significant enough to alter the TME. An unique characteristic of our study is that we used a mouse model where the biosynthesis of gangliosides is only modified in the tumor. Thus, fibroblasts, stroma, blood vessels and other immune cells in the TME, are still sialylated and therefore, might compensate for the lack of ST3Gal5 from tumor cells (Fig. S3E). This could be either by shedding of gangliosides directly acting on immune cells, or by transfer of the shed GSLs to cancer cells.

Shedding of gangliosides by tumor cells has immunomodulatory properties, suggesting that gangliosides may play an important role in contributing to an immunosuppressive TME (van der Haar Àvila et al. 2023). In both our mouse CRC cell lines, GD1a is the most abundant ganglioside (Table S1). This is also the case for glioblastoma cell lines, where levels of GD1a and GM2 were highly elevated and strongly promoted apoptosis of T cells (Chahlavi et al. 2005). Also, GD1a has the ability to reduce the DC stimulatory capacity in vitro, resulting in an impaired activation of CD4^+^ T cells (Caldwell et al. 2003), as well as skewing the differentiation of T cells towards a Th2 phenotype, favoring the development of Tregs (Jales et al. 2011). Besides, GD1a can be a potent inhibitor of several cytokines, such as IFNγ, TNFα, IL-6, IL-12, produced by T cells, macrophages and monocytes (van der Haar Àvila et al. 2023).

However, in line with the in vivo tumor growth data, we did not observe any significant differences in CD8^+^ T cell infiltration in MC38 and CT26 tumors with ST3Gal5-deficiency compared to the MOCK control tumors. Contrary to our results, ST3Gal5 positively correlated with CD8^+^ T cell infiltration and exhaustion in the TME of renal cell carcinoma tumors, indicated by CD8 and PD-1 co-localization in immunofluorescence stainings (Liu et al. 2022). Despite that, we found that CT26 tumors lacking gangliosides had significantly higher infiltration of CD4^+^ T cells and more specifically, increased numbers of Tregs in the TME. Tregs, characterized by the expression of FoxP3, are an immunosuppressive subset of CD4^+^ T cells that play a key role in maintaining self-tolerance. In the context of cancer, they are known to suppress anti-tumor immunity by several mechanisms, thereby promoting tumor development and progression (Togashi et al. 2019). Nevertheless, the increase in Tregs in ST3Gal5 KO tumors did not impact tumor growth in our CRC mouse model. An explanation for this could be that the immunosuppressive capacity of Tregs is counteracted by the high infiltration of CD8^+^ T cells in the TME. Additionally, Nagafuku *et al.* reported that activation of CD8^+^ and CD4^+^ T cells requires different types of gangliosides (Nagafuku et al. 2012). Distinct abundances of gangliosides in the TME, perhaps in uniquely functional lipid rafts, could therefore define immune functions in each T cell subset, thereby skewing the CD4^+^ T cell population towards a more immunosuppressive phenotype. Strikingly, CD8^+^ T cell activation is not affected in ST3Gal5 KO mice, whereas activation of CD4^+^ T cells, that requires *a*-series gangliosides, is severely compromised in those mice (Nagafuku et al. 2012). However, which specific CD4^+^ T cells subsets are affected is not known yet.

Another factor to consider in our model system could be the interaction of sialylated GSLs with Siglec receptors. Siglecs display distinct binding specificities for Sias on glycoproteins and glycolipids based on the underlying glycan moieties (Crocker et al. 2007). Thus, the composition of gangliosides in the TME defines which Siglec receptors are able to bind the Sias and therefore, able to tune the immune cell response. However, the overall Siglec binding might be altered, as we observed a reduction in binding of Siglec-F and Siglec-G in CT26-ST3Gal5 KO cells (data not shown). Our ST3Gal5 KO tumor cell lines do not have sialylated GSLs, but they still express α2-3 Sias carried on glycoproteins, suggesting that the modulation of the anti-tumor immune response in CRC is more likely mediated by sialylation on proteins rather than sialylated GSLs. In a similar study with MC38 cells, complete removal of Sias resulted in increased tumor growth in vivo paired with reduced frequencies of CD8^+^ T cells and NK cells in the TME (Cornelissen et al. 2019). On the other hand, abrogation of the sialic acid transporter in a B16 melanoma mouse model reduced the frequencies of Tregs, favoring an immune-dependent response and a slower tumor growth in vivo (Perdicchio et al. 2016a). Taken together, we thus propose that only Sias on proteins have a relevant impact on anti-tumor immunity in this model.

Several studies have shown the relevance of ST3Gal5 in regulating migration and invasion of tumor cells. For instance, silencing ST3Gal5 in murine breast cancer cells resulted in reduced cell migration and invasion in vitro*,* and led to a reduction in lung metastasis in vivo. The mechanism underlying the suppression of breast cancer migration and invasion was attributed to the activation of the phosphoinositide-3 kinase (PI3K)/Akt pathway, and consequently the inhibition of nuclear factor of activated T cell (NFAT)1 expression, which regulates cell proliferation (Gu et al. 2008). Similarly, a double GM3 and GM2 synthase KO in oncogene-transformed fibroblasts resulted in diminished tumor growth compared to WT, despite their identical cell proliferation kinetics in vitro (Liu et al. 2010). Furthermore, angiogenesis and blood vessel formation was decreased in the double KO tumors in contrast to the WT tumors (Liu et al. 2014). In our study, we also observed a reduction in the number of blood vessels in MC38-ST3Gal5 KO tumors, however it did not impact tumor growth. Prior research revealed that endothelial cells forming the blood vessels in MC38 tumors were distinct from endothelial cells found in the blood vasculature of CT26 tumors (Bertrand et al. 2021), which might explain the differential effect of ST3Gal5 on blood vessel formation in our study. Besides, breast cancer tumors in a ST3Gal5 KO mice had heightened vascularization paired with significantly faster tumor growth and angiogenesis compared to WT mice (Suzuki et al. 2021), suggesting that gangliosides derived from endothelial cells and stroma are also critical regulators of blood vessel formation and tumor angiogenesis.

Notably, in our CRC mouse models, we performed subcutaneous injections of the tumor cells and did not address metastasis formation. Highly metastatic melanoma cells are deficient in ganglioside biosynthesis, yet when injected subcutaneously into rats, the primary tumor regained expression of common gangliosides (GM3, GM2, GD3 and GD2), whereas the cells that metastasized to the lungs were still lacking gangliosides and exhibited an accumulation of LacCer (Zebda et al. 1995). Probably, metastatic tumors have a distinct ganglioside repertoire compared to the primary tumor and it is thus also important to take the tumor location into consideration when evaluating the TME.

Although the immunomodulatory effects of gangliosides have been widely described (van der Haar Àvila et al. 2023), nearly all the previous studies use isolated GSLs and in vitro models. Therefore, we are the first ones to show that sialylated glycosphingolipids, originating from the tumor, may impact blood vessel formation in MC38 tumors but not in CT26 tumors. Besides, we show that knocking out ST3Gal5 in CT26 tumors results in an increase of Tregs in the TME with limited impact on tumor growth in vivo. In conclusion, we demonstrate that the contribution of only ST3Gal5 on anti-tumor immunity is modest. Nevertheless, in combination with other sialyltransferases, the effect of glycan-dependent immune evasion might be larger, since it is not one single modification, but multiple glycosylation changes occurring in each tumor type (Rodriguez et al. 2022). Overall, our data suggest that tumor-derived sialylated GSLs have a limited impact on tumor immunity and that gangliosides derived from other cells in the TME or sialylated glycoproteins may have a larger contribution in tumor immune evasion.

## Materials and methods

### Colorectal cancer cohort

The dataset based on the Guinney et al. paper (Guinney et al. 2015) was analyzed on the R2 Genomics Analysis and Visualization platform (https://hgserver2.amc.nl). The normalized raw expression data from 726 CRC patients, of different cohorts, with a known CMS status was ranked from low to high and divided into *ST3Gal5^high^* (*n* = 578) and *ST3Gal5^low^* (*n* = 148) groups based on the optimal cut-off expression. Both groups were used to analyze the relapse-free survival.

### CRISPR/Cas9 constructs

CRISPR/Cas9 constructs were made as described by Nature Protocols from Ran *et al.* (Ran et al. 2013). The following guide RNA (gRNA) strands for murine *ST3Gal5* were used: ST3Gal5#1 top strand: CACCGGCAATGACGCCGATCGT; bottom strand: AAACACGATCGGCGTCATTGCC; and ST3Gal5 KO#3 top strand: CACCCCACATCGAACTGGTTGA; bottom strand: AAACTCAACCAGTTCGATGTGG. The gRNA strands were phosphorylated and annealed prior to cloning in the pSpCas9 (BB)-2A-Puro plasmid, a gift from Feng Zhang (Addgene #62988). Cloning mixtures were treated with PlasmidSafe exonuclease (Epicentre Illumina) to digest residual linearized DNA and used for transformation in XL1-Blue Subcloning-Grade competent bacteria (Stratagene). The Nucleobond Xtra Midi kit (Macherey-Nagel) was used to purify the plasmids. Purified constructs were sequenced by BaseClear with the following primer (hU6-F): GAGGGCCTATTTCCCATGATT. Purified DNA constructs were stored at −20 °C until further use.

### Generation of MC38/CT26-ST3Gal5 KO cell lines

MC38 cells (kind gift from Prof. M. van Egmond, Amsterdam UMC, Amsterdam, the Netherlands) were cultured in DMEM supplemented with 10% heat inactivated fetal calf serum (FCS, Biowest), 1% penicillin and 1% streptomycin (pen/strep). CT26 cells (obtained from ATCC) were cultured in RPMI-1640 medium supplemented with 10% FCS, 1% glutamine and 1% pen/strep. Both cell lines were kept at 37 °C and 5% CO_2_. MC38 and CT26 cells (70%–80% confluency) were transfected with CRISPR/Cas9 constructs (3 μg per 6-well) targeting the *ST3Gal5* gene (MC38/CT26-ST3Gal5 KO) or an empty CRISPR/Cas9 construct (MC38/CT26-MOCK). Lipofectamine LTX with PLUS™ reagent (Thermo Fisher Scientific) in Opti-MEM was used for transfection and applied according to the manufacturer’s protocol. After 24 h, fresh medium containing 6 μg/mL of puromycin for MC38 and 10 μg/mL of puromycin for CT26 was added to select for the modified cells. Transfected cells were incubated with 0.5 μg/mL of biotinylated anti-LacCer antibody (clone T5A7 (Iwabuchi et al. 2015)) for 30 min on ice, washed with PBA (PBS with 0.5% BSA and 0.02% NaN_3_) and incubated with 0.5 μg/mL of streptavidin-AF488 (Life Technologies) for 30 min on ice. After incubation, cells were washed and sorted in bulk on LacCer positive cells with the BD FACSAria™ Fusion sorter.

### Flow cytometry

The biotinylated anti-LacCer antibody (T5A7) was used to confirm the lactosylceramide expression on the KO cell lines, as described above. The biotinylated plant lectins MAL-I and MAA-II (*M. amurensis I* and *II,* Vector Laboratories) were used to detect α2-3 Sias on *N*-glycans and *O*-glycans, respectively (Geisler and Jarvis 2011). MC38 and CT26 cells were incubated with 5 μg/mL of the lectin for 30 min at 37 °C in HBSS Mg^2+^/Ca^2+^ with 0.5% BSA. Cells were washed with HBSS and incubated with 0.5 μg/mL streptavidin-AF488 (Life Technologies) and 7-Aminoactinomycin D (7AAD) live dead marker (Thermo Fisher) for 30 min at 37°C. Finally, cells were washed and acquired on the BD LSRFortessa™ flow cytometer. Data was analyzed with FlowJo v10.

For immune cell profiling, cells were incubated with Fc block (2.4G2) and LIVE/DEAD™ Fixable Aqua Blue (Thermo Fisher), as a fixable viability dye, for 15 min at 4°C. Cells were subsequently stained for 30 min at 4°C and in the dark with the following markers: CD45-PerCP (30-F11; Biolegend), CD3-BUV805 (17A2; BD Biosciences), CD8b-BUV563 (H35-17.2; BD Biosciences), CD4-BUV395 (RM4-5; BD Biosciences) and NKp46-BV421 (29A1.4; Biolegend). To stain for FoxP3, cells were fixed and permeabilized according to the manufacturer’s protocol (Foxp3 Transcription Factor Staining Buffer Set, eBioscience) and incubated with FoxP3-PE antibody (3G3; BD Biosciences) for 30 min at 4°C. Samples were acquired on the Cytek spectral flow cytometer 5 L Aurora. Data was analyzed with SpectroFlo 5-Laser software.

### GSL-glycan preparation from cells

MC38/CT26-MOCK and MC38/CT26-ST3Gal5 KO cells were washed three times with PBS. Cell pellets were snap frozen in liquid nitrogen and stored at −70°C until further use. The extraction of GSLs and the preparation of GSL-glycan alditols from cells were performed in triplicate, as previously described with slight modifications (Zhang et al. 2020; Zhang et al. 2023). Briefly, 4 × 10^6^ cells were lysed in 200 μL of water. Prior to GSL extraction, 2 μL of 5 μM iGb3 in ethanol was added as a spiked internal standard to monitor sample preparation and normalize absolute quantification. Crude GSLs were extracted and dried under vacuum in an Eppendorf Concentrator 5301 (Eppendorf, Hamburg, Germany) at 30°C. The extracted GSLs were dissolved in 100 μL of methanol followed by an addition of 100 μL water before the solid phase extraction (SPE) purification on tC18 RP cartridges. Eluate was collected and dried in an Eppendorf Concentrator. Next, a mixture of EGCase I (12 mU, 2 μL), EGCase I buffer (4 μL) and water (34 μL) (pH 5.2) was added to each sample and incubated for 36 h at 37°C to release the glycan head group. The released glycans were further purified by tC18 RP cartridges. The flow-through and wash fractions were collected and dried again. GSL-glycans were reduced to alditols in 30 μL of sodium borohydride (500 mM) in potassium hydroxide (50 mM) for 3 h at 50°C. After desalting, the glycan alditols were collected by combining flow-through and eluate and dried at 30°C. A porous graphitized carbon SPE clean-up was performed to further purify the samples. The purified glycan alditols were re-suspended in 20 μL of water prior to PGC nano-LC-ESI-MS/MS analysis.

### Analysis of GSL-glycan alditols using PGC nano-LC-ESI-MS/MS

The analysis of glycan alditols was performed using PGC nano-LC-ESI-MS/MS. Measurements were performed on an Ultimate 3,000 U high performance liquid chromatography (HPLC) system (Thermo Fisher) equipped with a home-packed PGC trap column (5 μm Hypercarb, 320 μm × 30 mm) and a home-packed PGC nano-column (3 μm Hypercarb 100 μm × 150 mm) coupled to an amaZon ETD speed ion trap (Bruker, Bremen, Germany). To analyze glycans, 2 μL of samples were injected. Separation was achieved with a linear gradient from 1% to 57% mobile phase B over 70 min at a 0.6 μL/min flow rate. The column was held at a constant temperature of 35°C. Ionization was achieved using the nanoBooster source (Bruker) with a capillary voltage of 1,000 V applied, a dry gas temperature of 280°C at 3 L/min, and isopropanol-enriched nitrogen at 3 psi. MS spectra were acquired within an m/z range of 340–1,850 in enhanced mode using negative ion mode. The smart parameter setting was set to m/z 900. MS/MS spectra were recorded using the three highest intensity peaks. Glycan structures were assigned based on the known MS/MS fragmentation patterns in negative-ion mode (Karlsson et al. 2004), elution order, and general glycobiological knowledge, with help of Glycoworkbench (Ceroni et al. 2008) and Glycomod (Cooper et al. 2001) software. Relative quantification of individual glycans was performed by normalizing the total peak area of all glycans within one sample to 100%. To estimate the glycan amount per cell, glycan intensity was normalized to the intensity of the internal standard iGb3. Afterwards, assuming the complete release of glycans and similar response factors between released glycan and iGb3 standard, the number of glycans per cell was estimated. Structures are depicted according to the Consortium of Functional Glycomics (CFG).

### Proliferation assay

Cells (1×10^3^ cells/well) were seeded in 96-well plates in triplicates in 100 μL of medium containing 10% FCS. The plates were put in an incubator at 37°C and 5% CO_2_ for 24, 48 and 72 h. At the indicated time points, 10 μL of the CellTiter-Blue® Cell Viability dye (Promega) was added and incubated at 37°C for 3–4 h. The proliferation capacity of the cells was measured on the FLUOstar Galaxy (MTX Lab systems, excitation 560 nm, emission 590 nm).

### In vivo tumor experiments

C57BL/6 (for MC38) and BALB/c (for CT26) mice from Charles River were used at 6–8 weeks of age at the animal facility of Amsterdam UMC. For all in vivo experiments, an equal distribution of female and male mice was used. Experiments were performed in accordance with national and international guidelines and regulations. Per mouse, 2×10^5^ tumor cells for MC38, and 5×10^5^ cells for CT26 were injected subcutaneously in the flanks under isofluorane. Tumors were measured three times per week in a double-blinded manner using a tumor caliper. Total tumor volume was calculated using the formula 4/3 × π × *abc* (*a* = width of the tumor/2, *b* = length/2 and *c* = the average of *a* and *b*). Mice were sacrificed when the tumor reached a size between 1,500 and 2,000 mm^3^. The tumor was isolated and used for further experiments.

### qRT-PCR analysis

MC38 WT and CT26 WT cell-lysis and mRNA isolation was performed with the mRNA capture kit (Roche, Bazel, Switzerland) and cDNA synthesis was performed using the Reverse Transcription System Kit (Promega, Wisconsin, USA), according to manufacturer’s instructions. Synthesized cDNA samples together with the KAPA SYBR® FAST Universal 2X qPCR Master Mix and the specific qPCR primers (listed below) were used for each qRT-PCR reaction. qRT-PCR for individual genes was ran and analyzed on the CFX96 Real-Time PCR Detection System (BIORAD, California, USA), with all target gene expression levels normalized to GAPDH (*M. Musculus*) mRNA levels.

To distinguish between WT and CRISPR-modified *ST3Gal5* we designed specific primers annealing to the gRNA-modified site within the *ST3Gal5* gene. These primers were used to evaluate our CRISPR-modified cell and to analyze expression in tissue sections. For the modified cell lines the normalized amount of target mRNA was calculated 2^Ct(GAPDH)-Ct(target)^ (Fig. S1B). For analyzing the expression in tissue, multiple 5 μm-thick cryosections per tumor were cut using a Leica cryostat and kept on dry ice. TRIzol reagent (Invitrogen) was added to the frozen tissue samples before vortexing thoroughly to homogenize. Next, RNA was isolated according to the manufacturer’s protocol and the yield and purity of each sample was assessed using a NanoDrop spectrophotometer N60 (Implent™). The RNA integrity was assessed on a bleach-gel by checking for the presence of three distinct bands for 28S, 18S and 5.8S/5S ribosomal RNA as described by Aranda et al. (Aranda et al. 2012). Subsequently, 1 μg of RNA was used for cDNA synthesis using the Reverse Transcription System Kit (Promega™) with Oligo (dT)15 Primer according to the manufacturer’s instructions. Prior to qPCR analysis, the cDNA samples were diluted 5-fold in nuclease-free water. RT-PCR was conducted in a 384- wells plate. Each 10 μL reaction contained 5 μL of Fast SYBR™ Green 2X Master Mix (Applied Biosystems™), 2 μL of the diluted cDNA, 2.8 μL of DEPC water and 0.2 μL of 10 μM forward and reverse primer mix. Each qPCR reaction was performed in triplicate on a QuantStudio™ 5 Real-Time PCR System, 384-well (Applied Biosystems™) with the default settings. The relative expression level of ST3Gal5 was calculated for each sample with: ∆Ct = Ct (ST3Gal5) – mean Ct (ref. genes), with glyceraldehyde-3-phosphate dehydrogenase (*GAPDH*) reference gene.

The primers used were designed using the NCBI primer designing tool (https://www.ncbi.nlm.nih.gov/tools/primer-blast/). ST3Gal5 forward primer: 5′-TGCCAGGCTGACTTCATCAC-3′, reverse primer: 5′- GTTGACGCAGGAGATCATGG-3′; *ST3Gal5* WT gene forward primer: 5′- CCAGCCAAAGCACTTCAGGA-3′, reverse primer: 5′- GGCAATGACGCCGATCGTG-3′; GAPDH forward primer: 5′- CCTGCACCACCAACTGCTTAG-3′, reverse primer: 5′- CATGGACTGTGGTCATGAGCC-3′.

### Immunofluorescence staining of tumor tissue

Dissected tumors were embedded in Tissue-Tek® OCT medium (Sakura *4583*), snap-frozen in liquid nitrogen and stored at −70°C. 5 μm thick sections were cut on a Leica cryostat and air-dried for 20 min at RT. The sections were then fixed in acetone for 10 min and rehydrated in PBS for 10 min before blocking with 5% normal goat serum (NGS) and 5% BSA for 30 min at RT. Slices were stained with either biotinylated anti-CD8 (53.2.72, produced in house) or with unconjugated anti-CD31 (14-0311-82, Invitrogen) primary antibodies for 2 h at RT. Subsequently, the tumor slices were incubated with either Streptavidin (S21374, 5 μg/mL; Invitrogen) or a goat anti-rat IgG antibody (A-21247, 5 μg/mL; Invitrogen), both conjugated to AF647, for 1 h at RT. Finally, nuclei were counterstained with 0.5 μg/mL DAPI (Invitrogen; D1306) for 5 min at RT. Whole slide fluorescent scans were acquired using the Olympus V200 (Olympus) or the Vectra Polaris (PerkinElmer) microscopes with a 20X air objective. For analysis, QuPath was used (Bankhead et al. 2017) and tissue areas, without folding, were selected based on the DAPI signal. Areas exceeding a predefined fluorescence threshold for AF647 and a specific size threshold were identified and categorized as positive objects. The number of positive cells (CD8^+^) or vessels (CD31^+^) was then normalized based on the tumor area to calculate the densities.

### Statistical analysis

Statistical significance was tested using GraphPad Prism 9 by performing unpaired nonparametric *t*-tests and two-way ANOVA with multiple comparison tests. Recurrence free-survival was displayed in Kaplan–Meier curves and statistical significance was calculated by chi-squared test on the R2-platform. (^*^P < 0.05, ^*^^*^P < 0.01; ^*^^*^^*^P < 0.001; ^*^^*^^*^^*^P < 0.0001; ns: not significant).

## Supplementary Material

Supplementary_figures_cwae036

## Data Availability

The data that support the findings of this study are available from the corresponding author, S.J. van Vliet, upon reasonable request.
