# Supplementary material for: Limited impact of cancer-derived gangliosides on anti-tumor immunity in colorectal cancer
Source: Glycobiology. 2024 May 24;34(7):cwae036. doi: 10.1093/glycob/cwae036 (PMC11137322; doi:10.1093/glycob/cwae036)
Supplement: Supplementary_figures_cwae036 [file supplementary_figures_cwae036.docx]

**Supplementary figures**


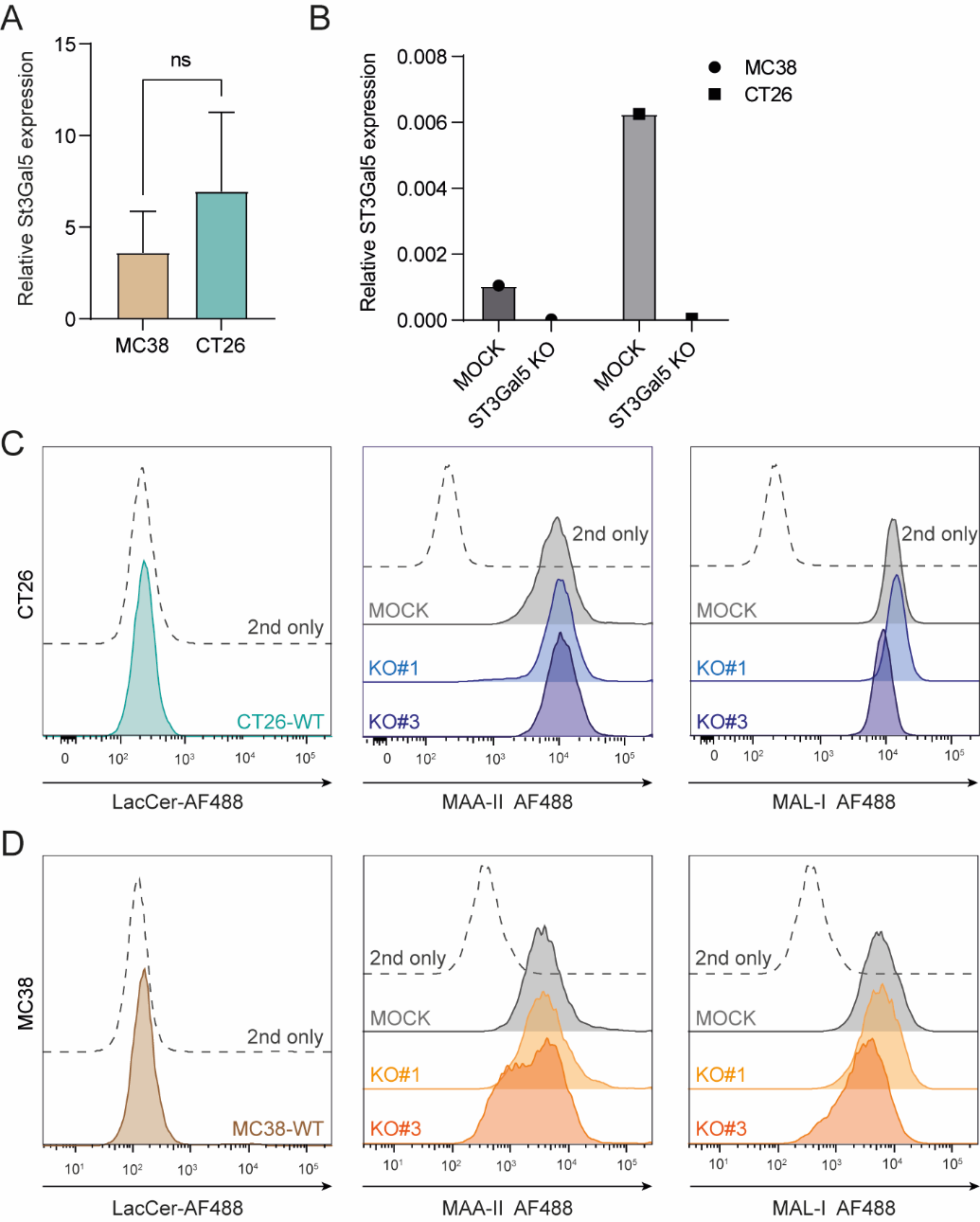


**Figure S1. ST3Gal5 KO does not affect sialylation of glycoproteins. A** *ST3Gal5* expression relative to GAPDH from MC38 and CT26-WT cells, by qRT-PCR. Mean ± SD; unpaired non-parametric *t-*test (ns, not significant). **B** ST3Gal5 expression relative to GAPDH from MOCK and ST3Gal5 KO cell lines, by qRT-PCR. **C, D** CT26 (**C**) and MC38 (**D**) WT cells do not express LacCer on the cell surface. α2-3 sialic acids on *N*- and *O*-glycoproteins of CT26 (**C**) and MC38 (**D**) cells were measured by flow cytometry using biotinylated plant lectins MAL-I and MAA-II, respectively, and with Streptavidin conjugated to AF488. MOCK-transfected cells were used as a positive control.

**
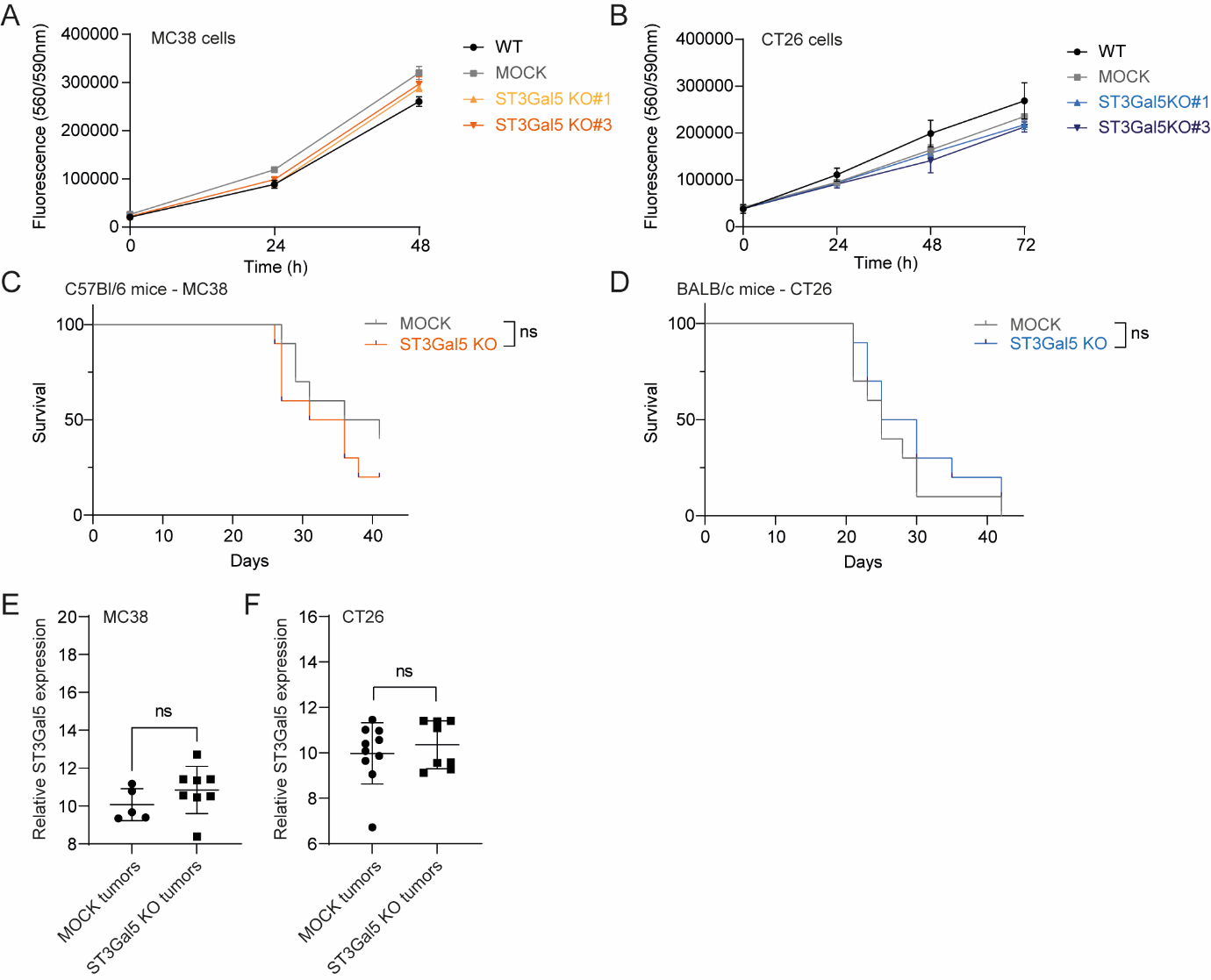
**

**Figure S2. Similar proliferation rates and *in vivo* survival of ST3Gal5 KO and MOCK cells*.*** The proliferation capacity of MC38 (**A**) and CT26 (**B**) MOCK and ST3Gal5 cells was tested in triplicates with CellTiter-blue^®^ Cell Viability dye and the fluorescence intensity was measured after 24, 48, and 72h. Mean ± SD. **C**,**D** MOCK or ST3Gal5 KO cells were injected subcutaneously into C57Bl/6 mice (for MC38 cells) and into BALB/c mice (for CT26 cells). Mice were sacrificed when the tumors reached a size between 1500 mm^3^ and 2000 mm^3^. Survival curves for MC38 (**C**) and CT26 (**D**) are shown (ns, not significant). **E**,**F** *ST3Gal5* expression relative to GAPDH from MC38 (**E**) and CT26 (**F**) frozen tumor tissues was assessed by RT-qPCR using primers specific for the unmodified *ST3Gal5* gene. Mean ± SD; unpaired non-parametric *t-*test (ns, not significant).

**
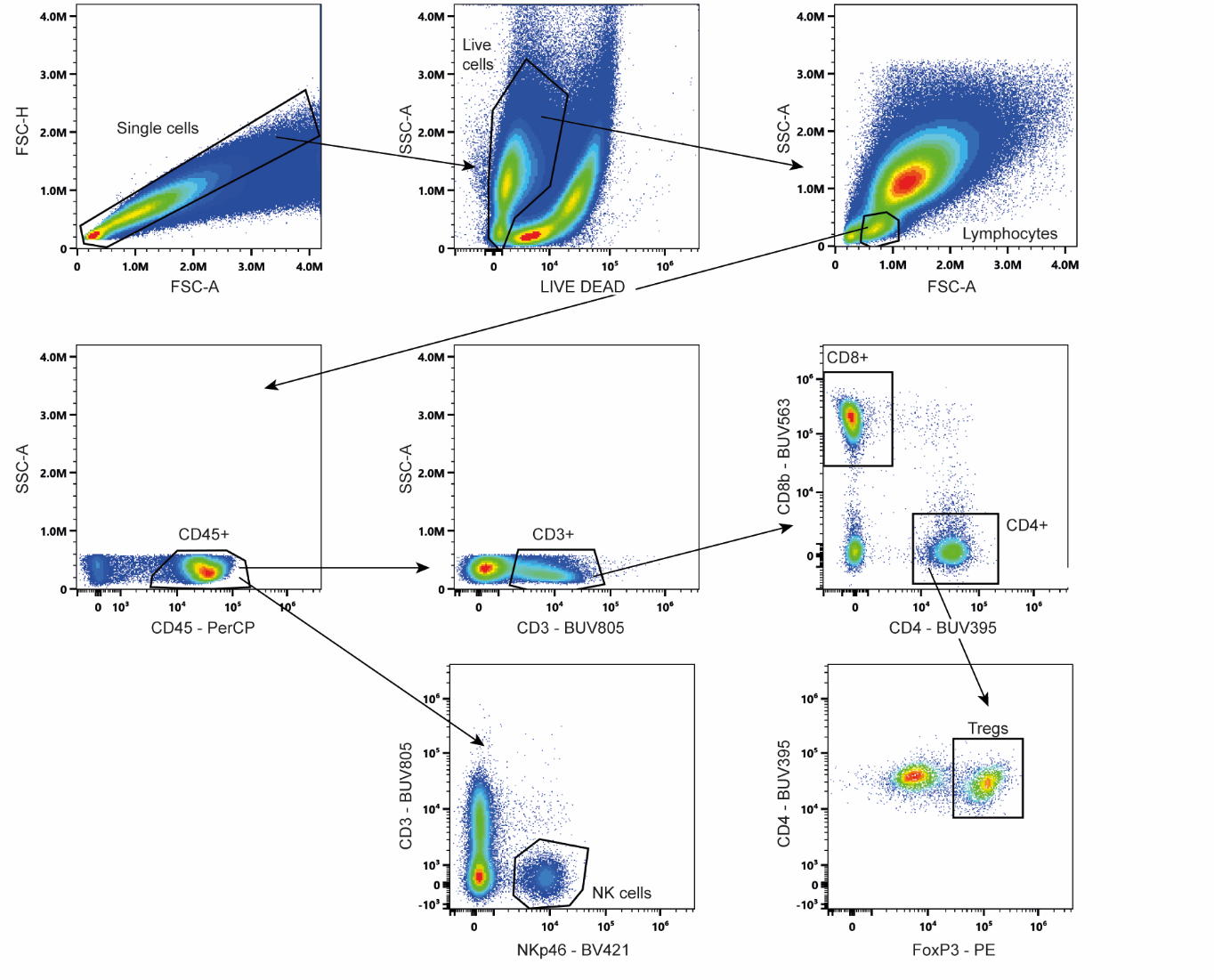
**

**Figure S3. Manual gating strategy of one representative CT26 tumor cell suspension.** All samples were analyzed by flow cytometry and gated on respectively single cells, live cells and lymphocytes (based on the FSC and SSC) (Top row plots). From the lymphocyte gate, we gated and CD45^+^ immune cells (middle row left plot). T cells were further gated from the CD45^+^ gate on CD3^+^ cells and subgated on CD4^+^ and CD8^+^ T cells (middle row, middle and right plot). Tregs were gated from the CD4^+^ T cells as FOXP3^+^ cells (bottom row, right plot). NK cells were gated from the CD45^+^ population, being CD3^-^ and NKp46^+^ (bottom row left plot). Arrows indicate the order of gating.

**Table SI. List of GSLs detected on MC38 and CT26 cells.** Glycan structures were assigned based on MS/MS fragmentation and glycobiological pathway constraints. Structures are depicted according to the CFG (Consortium of Functional Glycomics) nomenclature. Blue square: *N*-acetylglucosamine, yellow square: *N*-acetylgalactosamine, blue circle: glucose, yellow circle: galactose, red triangle: fucose, pink diamond: *N*-acetylneuraminic acid, grey diamond: *N*-glycolylneuraminic acid. a, b: isomer number; SD: standard deviation; n.d.: not detected. Expression of gangliosides is shown as percentage of the relative abundance within each cell type.

**Table SI**

| **Glycan number** | **Glycan name** | **Proposed structure** | **Relative abundance % (SD %)** | | | | **Theoretical** | **Observed** | **Deviation** | **Matched fragments for MS/MS** |
| --- | --- | --- | --- | --- | --- | --- | --- | --- | --- | --- |
|  |  |  | MC38  Control | MC38  ST3GAL5 KO | CT26  Control | CT26  ST3GAL5 KO | [M-H]^-^ | [M-H]^-^ | Δ[M-H]^-^ |  |
| 1 | Lc2 | 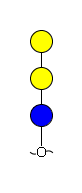 | 0.41 (±0.35) | 0.15 (±0.09) | n.d. | n.d. | 343.12 | 343.05 | 0.07 | 4 |
| 2 | GM3 | 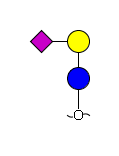 | 7.44 (±0.36) | 2.91 (±1.17) | 2.87 (±0.22) | 4.08 (±0.59) | 634.22 | 634.24 | 0.02 | 3 |
| 3 | GM2 | 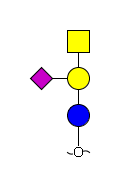 | 14.70 (±3.90) | 5.64 (±1.44) | 22.20 (±2.63) | 3.85 (±0.65) | 837.30 | 837.36 | 0.06 | 8 |
| 4 | GM1a | 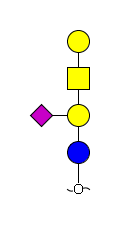 | 21.01 (±1.35) | 2.63 (±0.92) | 28.52 (±2.94) | 3.12 (±0.48) | 999.35 | 999.42 | 0.07 | 14 |
| 5 | GD1a | 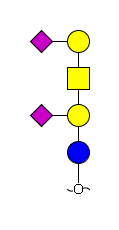 | 53.45 (±1.76) | 4.68 (±1.45) | 42.85  (±3.26) | 5.72 (±0.33) | 1290.45 | 1290.49 | 0.04 | 8 |
| 6 | GD1α | 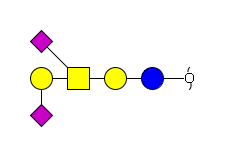 | 1.24 (±0.15) | 20.37 (±1.25) | 1.11 (±0.27) | 14.37  (±2.11) | 1290.45 | 1290.38 | 0.07 | 8 |
| 7 | GM3-Neu5Gc | 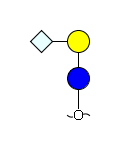 | 0.56 (±0.21) | 1.49 (±0.79) | 0.29 (±0.06) | 1.65 (±0.24) | 650.21 | 650.26 | 0.05 | 5 |
| 8 | GM2-Neu5Gc | 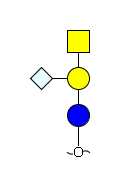 | 0.28 (±0.11) | 0.93 (±0.30) | 0.37  (±0.07) | 0.67 (±0.07) | 853.29 | 853.43 | 0.14 | 4 |
| 9 | GD3 | 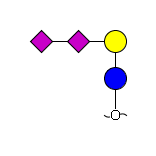 | 0.90 (±0.30) | 3.12 (±1.41) | 0.59 (±0.16) | 2.94 (±0.03) | 925.31 | 925.36 | 0.05 | 6 |
| 10 | Gg3 | 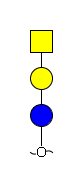 | n.d. | 0.96 (±0.22) | 0.27 (±0.05) | 4.88 (±0.24) | 546.20 | 546.23 | 0.03 | 7 |
| 11 | Gg4 | 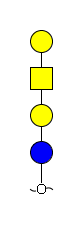 | n.d. | 17.09 (±5.37) | 0.51 (±0.09) | 23.88 (±3.64) | 708.26 | 708.31 | 0.05 | 8 |
| 12 | GM1b | 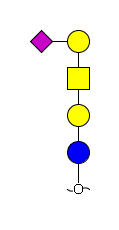 | n.d. | 32.15  (±5.19) | n.d. | 25.13 (±1.60) | 999.35 | 999.43 | 0.08 | 10 |
| 13 | GM1α | 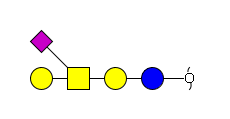 | n.d. | 0.13 (±0.05) | n.d. | 6.94 (±0.82) | 999.35 | 999.40 | 0.05 | 17 |
| 14 | Gal-nLc4 | 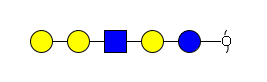 | n.d. | n.d. | 0.28  (±0.13) | 1.83 (±0.33) | 870.31 | 708.40 | 0.09 | 14 |
| 15 | S(3)nLc6 | 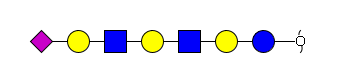 | n.d. | 1.18 (±0.29) | 0.16 (±0.02) | 0.91 (±0.08) | 1364.48 | 1364.51 | 0.03 | 12 |
